# Body mass index and abdominal waist values are related to increased cardiometabolic risk in schoolchildren aged five to ten years

**DOI:** 10.1590/1984-0462/2024/42/2022113

**Published:** 2023-07-10

**Authors:** Maria de Fatima de Oliveira, Ariana Rodrigues da Silva Carvalho, Bruna Schumaker Siqueira, Bruna Emília Mareco de Almeida, Claudia Silveira Viera, Gicelle Galvan Machineski, Beatriz Rosana Gonsalves de Oliveira Toso, Sabrina Grassiolli

**Affiliations:** a Colégio Estadual Jardim Consolata, Cascavel, PR, Brazil.; b Universidade Estadual do Oeste do Paraná, Cascavel, PR, Brazil.; c Hospital São Lucas, Cascavel, PR, Brazil.

**Keywords:** Students, Obesity, Cardiovascular risk, Metabolism, Estudantes, Obesidade, Risco cardiovascular, Metabolismo

## Abstract

**Objective:**

To evaluate the frequency of obesity and cardiometabolic risk in schoolchildren under ten years old.

**Methods:**

This is a cross-sectional study with schoolchildren (n=639) aged five to ten years in a municipally of southern of Brazil. The cardiometabolic risk was calculated from values of body mass index (BMI), waist circumference (WC), diastolic (DBP) and systolic blood pressure (SBP), blood glucose levels, triglycerides and total cholesterol (TC). Odds ratio (OR), Spearman correlation and principal component analysis (PCA) were analyzed.

**Results:**

Independent of sex, elevated WC and BMI were related to higher values of SBP, DBP, and TC in schoolchildren. The frequency of cardiometabolic risk was 6.0% in girls and 9.9% in boys. Schoolchildren with elevated values of SBP, triglycerides and TC presented high OR for cardiometabolic risk. PCA indicated that schoolchildren with high WC (p>80) presented more frequently altered glucose levels, triglycerides, and TC.

**Conclusions:**

Obesity, especially when associated with elevated WC, is related to metabolic dysfunctions and cardiometabolic risk in schoolchildren under ten years of age. These findings indicate the urgency of stablishing metabolic risk for this age group, enabling early diagnosis and adequate treatment, to prevent the development of diabetes and cardiovascular dysfunction throughout life.

## RESUMO

**Objetivo:**

Avaliar a frequência de obesidade e risco cardiometabólico em escolares menores de dez anos de idade.

**Métodos:**

Este é um estudo transversal com escolares (n=639) com idade de cinco a dez anos de um município do Sul do Brasil. O risco cardiometabólico foi calculado com base nos valores do índice de massa corpórea (IMC), circunferência da cintura (CC), pressão arterial sistólica (PAS) e diastólica (PAD), valores sanguíneos de glicose, triglicerídeos e colesterol total. *Odds ratio* (OR), correlação de Spearman e análise de componentes principais (PCA) foram obtidos.

**Resultados:**

Independentemente do sexo, CC e IMC aumentados foram relacionados com maiores valores de PAS, PAD e colesterol total. A frequência de risco cardiometabólico foi de 6,0% nas meninas e 9,9% nos meninos. Escolares com elevados valores de PAS, triglicerídeos e colesterol total tinham alto OR para risco cardiometabólico. A PCA mostrou que escolares com alta CC (p>80) apresentam mais frequentemente alterações nos níveis sanguíneos de glicose, triglicerídeos e colesterol total.

**Conclusões:**

A obesidade, especialmente quando associada a elevados valores de CC, está relacionada com disfunções metabólicas e risco cardiometabólico em escolares menores de dez anos de idade. Estes achados indicam a urgência de estabelecer o risco metabólico para essa faixa etária, possibilitando o diagnóstico precoce, o adequado tratamento para evitar o desenvolvimento de diabetes e disfunções cardiovasculares ao longo da vida.

## INTRODUCTION

In four decades (1975–2016), the number of obese children and adolescents increased ten times worldwide including in Brazil.^1,2^ In childhood, such as in adult life, the excessive white adipose tissue (WAT) elevates the risk of developing non-communicable chronic diseases, in particular type 2 diabetes and cardiovascular diseases.^3^ The accelerated WAT expansion contributes to the onset of insulin resistance and disruptions of glucose, lipid and cardiovascular homeostasis, a process related to high levels of pro-inflammatory cytokines released by hypertrophied adipocytes that define the metabolic syndrome (MS).^4,5^ Despite overweight and obesity being evident problems in children and adolescents, in this stage of life, the characterization and cut points of the MS are undefined, in contrast to the adult obese population.^6,7^

In Brazil, public reports consolidated through an electronic address, named *Sistema de Vigilância Alimentar e Nutricional* (Sisvan Web) by the Ministry of Health, showed that, in 2019, 7.8% of under five-year-old children and 10.1% of five to ten-year-old children are overweight. Childhood obesity is frequently associated with hyperinsulinemia, dyslipidemia, hypertension, sleep apnea, gastrointestinal, musculoskeletal and orthopedic complications, besides the accelerated onset of cardiovascular diseases and type 2 diabetes throughout life.^8,9^ Thus, as emphasized by important review studies, it is urgent to establish cut-points considering age, sex and pubertal stage, for hormonal, blood biochemistry and cardiovascular variables, in order to estimate cardiometabolic risk in children under ten years old.^9–11^

The International Diabetes Federation defined MS in children and adolescents aged 10 to 16 years using the following criteria: WC above the percentile 90^th^ (p>90) and alteration in two or more variables; fasting glucose >100 mg/dL; triglycerides >150 mg/dL: high density lipids (HDL) <40 mg/dL; and systolic blood pressure (SBP) >P95.^6^ Based on the definition of the International Diabetes Federation, a Brazilian research named *Estudo de Riscos Cardiovasculares em Adolescentes* (ERICA) assessed the prevalence of factors related to MS in more than 37 thousand Brazilian students between 12 and 17 years-old. The results pointed out a 2.6% MS prevalence in Brazilian children, especially in the southern region of Brazil and in students of the public educational system.^10^ Except for one study, there is no other research exploring cardiometabolic risk in Brazilian children under ten years old. Thus, the objective of the present study was to evaluate the frequency of obesity and cardiometabolic risk among schoolchildren, from five to ten years-old.^11^

## METHOD

This is a cross-sectional study conducted on schoolchildren from a city of Western Parana State, Brazil. All procedures involving these students were approved by the Ethics Committee of the Western Parana State University (UNIOESTE), Cascavel, Paraná (PR), protocol number 1.872.666 (CAAE 60942716.8.0000.0107), and all regulations of the Resolution 466/2012 of the National Health Council (2012) were met. The following inclusion criteria were considered: girls and boys between 5–10 years old (n=639), regularly enrolled in two municipal elementary schools.

Anthropometric and cardiovascular variables, besides the blood analysis of each child were performed by trained nurses and nursing students. The obtained values and sociodemographic information were registered in a survey questionnaire designed exclusively for this study.

The assessments of body weight and height of the children were performed according to the guidelines established by the Brazilian Ministry of Health.^12^ During the anthropometric measures, the child wore light clothes and remained shoeless, being adequately positioned. The body weight (kg) was evaluated through a calibrated anthropometric mechanical balance (Omron® 1 up to 150 kg) and the height (m) was registered by using a portable stadiometer (Avanutri®; 20 up to 200cm). Height and body weight were used to obtain BMI (kg/m^2^), according to the Z score cutoff recommended by the World Health Organization (WHO).^13^

A non-elastic flexible tape (WCS®) was used to verify the WC (cm), which was positioned horizontally at the level of the navel, midway between lowest rib and the iliac crest. Considering that the present study evaluated schoolchildren under ten years of age, the cutoff to WC was defined based on a recent published study with the same population, by Santos et al.^14^ Thus, WC was considered high when the value was above the 80^th^ percentile (P>80), according to sex and age.

The systolic and diastolic blood pressure (SBP and DBP; mmHg) and heart frequency (HF; bpm) were measured through a digital automatic blood pressure monitor (Omron®) using the child’s left wrist, in a sitting position, after 15 minutes of rest. At least, two measurements were performed at a five-minutes interval. The cutoff to SBP and DBP were defined as recommended by the 7^th^ Brazilian Guideline of Arterial Hypertension.^15^ Thereby, the SBP and DBP were considered high when the value was within or above the 90th percentile (p≥90).

The glucose, triglycerides and total cholesterol (TC) were analyzed in non-fasting condition, using the fresh capillary whole blood obtained from peripheral finger puncture through automatic sterile lancets (Premium®). The blood was immediately transferred to appropriate test strips, making use of the glucose meters (Accu-Chek®), in order to register the glucose level. Afterwards, the triglycerides and TC values were registered with Accutrend PlusMeters® (Roche Diagnostics, North America). The glucose was considered high when the level was greater than 140mg/dL, according to what is proposed by the International Diabetes Federation for children (10–16 years old) in non-fasting condition.^16^ High values of triglycerides (≥101 mg/dL) and TC (≥171 mg/dL) were defined by the Brazilian Consensus for the Normalization of Laboratory Determination of Lipid Profile for children aged 10–19 years-old.^17^

Herein, we adopted the concept of cardiometabolic risk, as suggested by other studies, to describe a cluster of metabolic alterations associated with obesity in children under ten years old.^18,19^ For this, those schoolchildren who presented WC p>80 were considered at cardiometabolic risk when associated with at least two elevated values of the following variables: glucose (>140 mg/dL); triglycerides (≥101 mg/dL); TC (≥171 mg/dL); SBP and DBP (p≥90).

Statistical analysis: Analyses were performed by the software R (R Development Core Team, 2018). Qui-square or Monte-Carlo test were used to evaluate sex effects. Quantitative variables were analyzed by Students *t*-test or Mann-Whitney test, after checking normality (Shapiro-Wilk) and homoscedasticity (F test) assumptions. The OR for cardiometabolic risk was evaluated through mathematical model by binary logistic regression with goodness of fit measured by the Hosmer & Lemeshow. The Spearman correlation was used to analyze the relationship between elevated WC and BMI and alterations in blood biochemistry values and pressure variables. Finally, the schoolchildren were grouped according to sex and WC (p<80 and p>80) and these categories submitted to PCA with two-way ANOVA and Tukey-HSD post-test to compare the groups. In all analyses it was considered the p-value (p<0.05).

## RESULTS

The Table 1 represents anthropometric, cardiovascular, and metabolic parameters comparing girls and boys between 5–10 years old. Despite the similar height observed between the sexes, the boys showed higher values of body weight (p=0.030), WC (p=0.005), and TC (p=0.007) levels. The HF were slightly elevated in girls when compared with boys (p=0.045). The SBP, DBP, glucose and triglycerides blood levels were similar between both sexex.

**Table 1.** Anthropometric, cardiovascular pressure and blood biochemistry variables in five to ten years old girls and boys.

	Girls (n=269)		Boys (n=244)		p-value*
	Mean±SD	min-max	Mean±SD	min-max	
Body weight (kg)	28.1±8.8	15.6–64.3	29.5±9.0	17.2–71.9	0.030
Height (cm)	1.3±0.1	1.0–1.6	1.3±0.1	1.0–1.6	0.134
WC (cm)	59.1±8.4	45.0–91.0	61.0±9.0	48.0–105.0	0.005
DBP (mmHg)	64.7±11.7	40.0–137.5	64.2±11.1	33.0–103.0	0.846
SBP (mmHg)	99.7±12.4	70.0–166.0	100.7±12.5	61.0–148.0	0.093
HF (rpm/min)	91.3±12.8	62.0–127.0	88.5±13.1	15.2–131.0	0.045
Glucose (mg/dL)	96.5±16.3	65.0–164.0	97.9±16.5	52.0–166.0	0.175
Triglycerides (mg/dL)	66.6±64.1	35.0–461.0	64.9±54.8	35.0–347.0	0.793
Total cholesterol (mg/dL)	104.1±43.2	75.0–215.0	116.2±50.1	75.0–265.0	0.007

SD: standard deviation; WC: waist circumference; DBP: diastolic blood pressure; SBP: systolic blood pressure; HF: heart frequency. *p-value were obtained for Student’s *t*-test or Mann-Whitney test. Values in bold mean statistical difference p<0.05.

Girls and boys showed similar frequency of elevated values of WC, DBP and SBP. However, the frequency of high values of TC were significantly greater in boys (17.7%) compared to girls (9.2%); (Table 2, p<0.006).

**Table 2.** Frequency of high values of waist circumference, cardiovascular blood pressure and blood biochemistry parameters in five to ten years old schoolchildren.

	Girls		Boys		p-value*
	Normal n (%)	High n (%)	Normal n (%)	High n (%)	
WC (cm)	197 (79.1)	52 (20.9)	170 (73.3)	62 (26.7)	0.132
DBP (mmHg)	183 (73.5)	66 (26.5)	161 (69.4)	71 (30.6)	0.320
SBP (mmHg)	205 (82.3)	44 (17.7)	195 (84.1)	37 (16.0)	0.614
Glucose (mg/dL)	244 (98.0)	5 (2.0)	228 (98.3)	4 (1.7)	0.818
Triglycerides (mg/dL)	203 (81.5)	46 (18.5)	188 (81.0)	44 (19.0)	0.890
Total cholesterol (mg/dL)	226 (90.8)	23 (9.2)	191 (82.3)	41 (17.7)	0.006

WC: waist circumference; DBP: diastolic blood pressure; SBP: systolic blood pressure. *p-value were evaluated by Chi Square Test. Values in bold mean statistical difference p<0.05.

As indicated in Table 3, the BMI Z score categorization was also influenced by sex. The relative frequency of eutrophic children was significantly higher in girls (69.4%) in relation to boys (55.3%); p<0.004. However, the relative frequency of underweight, overweight, and obese distribution was similar between them (p>0.05).

**Table 3.** Categorization of Z score of body mass index and cardiometabolic risk of five to 10 years old girls and boys.

Z score BMI	Girls n (%)	Boys n (%)	p-value
Underweight (Z score < -2)	2 (0.8)	7 (2.9)	0.004*
Eutrophic (-2 ≤ Z score < 1)	186 (69.4)	135 (55.3)	
Overweight (1≤ Z score < 2)	50 (18.7)	60 (24.6)	
Obese (Z score ≥2)	30 (11.2)	42 (17.2)	
Cardiometabolic risk			
No	234 (94.0)	209 (90.1)	0.114^†^
Yes	15 (6.0)	23 (9.9)	

BMI: body mass index. *Monte-Carlo; ^†^Chi-square test to independent analysis.

**Figure 1.**
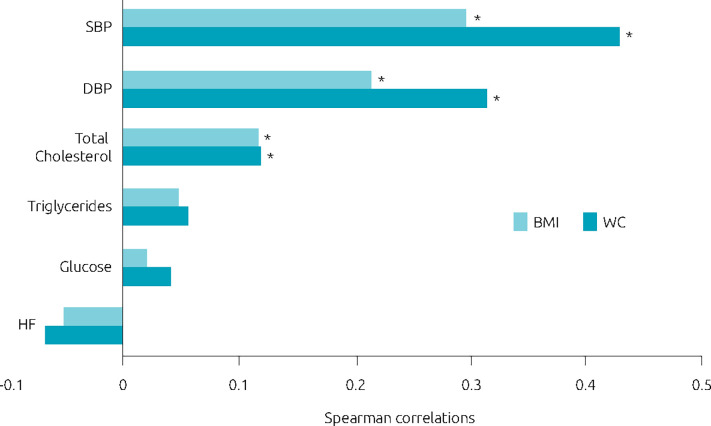
Association between waist circumference and body mass index with metabolic and pressure variables in scholar children.

Figure 1 shows the values of the Spearman correlation coefficient (r) between the BMI and WC with pressure and metabolic variables in scholar children. High values of WC and BMI Z score were positively correlated to elevated values of SBP; DBP and TC in scholar children; being WC the strongest association. Therefore, high WC in scholar children were significantly (p<0.05) associated with elevated values of SBP (r>0.4), DBP (r>0.3) and TC (r>0.1).

Waist circumference and body mass index Spearman correlations rank with variables triglycerides, total cholesterol, glucose, heart frequency, systolic blood pressure and diastolic blood pressure in scholar children independent of sex. *p<0.05 SBP: systolic blood pressure; DBP: diastolic blood pressure; HF: heart frequency; BMI: body mass index; WC: waist circumference.

The frequency of cardiometabolic risk was similar between the sexes, being 6.0% in girls and 9.9% in boys (Table 3; p=0.114). The OR for cardiometabolic risk is showed in Table 4. Regardless of sex, elevated OR for cardiometabolic risk was observed among schoolchildren with high values of SBP (OR 7.35 [2.85–18.95]; p<0.001), triglycerides (OR 6.33 [2.44–16.48; p<0.001); and TC (OR 30.12 [9.23–98.28]; p<0.0001). On the other hand, the adequate values of DBP were related with significant reduction in OR for cardiometabolic risk (OR 0.02 [0.01–0.09]; p<0.001), and altered glucose levels were not related with high OR for cardiometabolic risk (OR 8.34 [0.51–136.36]; p<0.137).

**Table 4.** Odds ratio for cardiometabolic risk in five to ten years old schoolchildren.

	Classification	Value	p-value	OR (95%CI)
Intercept	–	-3.10	<0.001	–
Sex	Boys	0.00	–	–
	Girls	0.00	–	–
SBP (percentile)	Adequate (<P90)	0.00	–	–
	High (≥P90)	2.0	<0.001	7.35 [2.85–18.95]
DBP (percentile)	Adequate (<P90)	-3.72	<0.001	0.02 [0.01–0.09]
	High (≥P90)	0.00	–	–
Triglycerides (mg/dL)	Adequate (<101)	0.00	–	–
	High (≥101)	1.85	<0.001	6.33 [2.44–16.48]
Total cholesterol (mg/dL)	Adequate (≤170)	0.00	–	–
	High (>170)	3.41	<0.001	30.12 [9.23–98.28]
Glycemia (mg/dL)	Adequate (<140)	0.00	–	–
	High (≥140)	2.12	0.137	8.34 [0.51–136.36]

OR: odds ratio; 95%CI: 95% confidence interval; SBP: systolic blood pressure; DBP: diastolic blood pressure.

**Figure 2.**
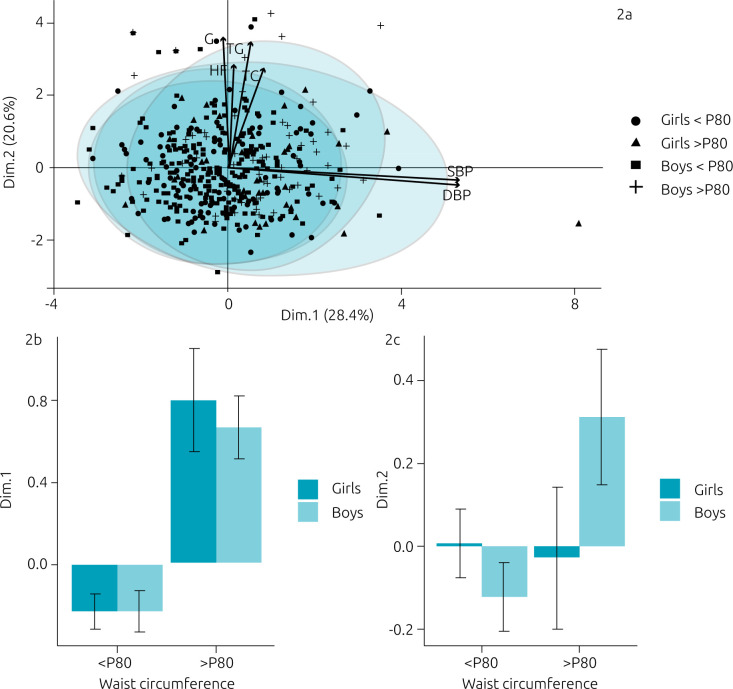
Principal analysis component.

Through PCA, we compared metabolic and pressure variables in girls and

boys, considering WC (p<80 versus p>80) (Figure 2a). The extensive overlap of data suggests many similarities between the sexes, with two central axis or dimensions (Dim) explaining the greater data variance (49%). The Dim.1 (Figure 2b) is represented by variables heart frequency (HF), triglycerides (TG), total cholesterol (TC), and glucose (G) and contributes with 28.4% (auto-value=1.70) of data variance. The Dim.1 showed a significant effect of WC percentile (F_1_=47.52; p=0.000): schoolchildren with elevated WC p>80 presenting greater values of HF, TG, TC and G, compared to schoolchildren with WC p<80 (Figure 2b). This effect was independently of sexes (F_1_=0.135; p=0.710).

The principal component analysis (Figure 2a) shows the variables: triglycerides, total cholesterol, glucose, heart frequency, systolic blood pressure and diastolic blood pressure, categorized according to sex (boys and girls) and waist circumference (p<80 and p>80). The Dim.1 (Figure 2b) represents the variables triglycerides, total cholesterol, glucose and heart frequency while the Dim.2 (Figure 2c) represents the variables systolic blood pressure and diastolic blood pressure. TG: triglycerides; TC: total cholesterol; G: glucose; HF: heart frequency; SBP: systolic blood pressure; DBP: diastolic blood pressure.

The Dim.2 is represented by SBP and DBP variables, which explains 20.6% (auto-value=1.24) of the data variance. Neither WC percentile (F_1_=3.14; p=0.080) nor sex factor (F_1_=0.00; p=0.100) influenced the variables in Dim.2, and there was no interaction (F_1,434_=3.54; p=0.060) between the sex and WC percentiles in this Dim (Figure 2c).

## DISCUSSION

The main findings of the present study showed that schoolchildren between 5–10 years of age have a high frequency of overweight and obesity, with increased WC and cardiometabolic risk. The excessive WAT in children and adolescents is frequently related to endocrine-metabolic dysfunctions such as hyperinsulinemia, insulin resistance, dyslipidemia, glucose intolerance, and cardiovascular abnormalities, a condition very similar to those observed in obese adults with MS. However, in childhood the definition of MS is very inconsistent, especially among children under ten years old.^8,18,19^

In 2017, the Lancet journal published the global estimative of obesity prevalence in children and adolescent from 1975 to 2016. This report demonstrated that overweight and obesity in this population remain increased, mainly in low- and middle-income countries.^2^ Meantime, the higher fluctuation in BMI was observed among children and adolescents compared to adults in Latin America. Herein, applying Z score of BMI, it was observed that 19% of girls and 25% of boys are overweight, and the frequency of obesity was 11.2% in girls and 17% in boys. Similar frequencies are registered by Sisvan Web of the Brazilian Ministry of Health, regarding 5–10 year old children, from the southern region of Brazil.

Sentalin et al., who evaluated obesity in Brazilian schoolchildren between 6–8 years old in southeastern region of Brazil also found similar obesity frequencies. Such values are above the Brazilian national median of overweight and obesity in children under ten years old.^11^ Above all, in our sample, regardless of sex or obesity, approximately 20–25% schoolchildren had high values of WC. Filgueiras et al. evaluated 4–9 years old children in the southeastern region of Brazil, and revealed 65% of high WC p>90 among obese children, pointing out a higher measure in girls. These data showed that the overweight and obesity rates are increasing in Brazilian children under ten years old.^20^

The metabolic blood parameters, evaluated along the present study, demonstrated that lipids had higher values among schoolchildren. More than 20% of these individuals had elevated values of triglycerides, suggesting that this lipid could be an important marker of disruption in metabolism among children under ten years old. In this regard, elevated triglycerides and glucose index (a marker of insulin resistance) was positively related with high WC and sedentarism in children aged 4–7 years, in the southeastern region of Brazil.^21^ Our data also demonstrated that higher values of TC were observed in boys, when compared to girls. Elevated frequency of hypercholesterolemia was also observed in obese children in the southeast of Brazil. In this study, 220 obese children were evaluated between 5–14 years old, of these, 40% of boys and 30% of girls showed elevated TC levels, with no sex effect.^22^

Hypertension also has been frequently observed in obese children.^23^ Moreover, around 20% of schoolchildren aged 5–10 years old had high values of SBP, and 30% had high values of DBP, indicating early alterations in cardiovascular pressure measures. There is a lack of evidence among Brazilian studies evaluating hypertension in children under 10 years old. In a systematic literature review, Magliano et al. indicated that among children and adolescents between 10–20 years old, in the southern and northeastern Brazilian regions, the prevalence of hypertension was approximately 8.1%.^24^

Similarly, the frequency of hypertension was registered by ERICA group in more than 73 thousand Brazilian adolescents aged 12–17 years in the south region. In this work, hypertension was found in 11.6% of girls and in 22.3% of boys.^9^ Although isolated measures of SBP and DBP, as done in the present study, are insufficient to define hypertension, mainly in children under ten, our data reinforce the need to establish pressure parameters for this infancy stage.

Herein, we found that 6.0% of girls and 9.9% of boys are at cardiometabolic risk. The ERICA group, considering the International Diabetes Federation’s definition of MS, found approximately 4.1% of MS in Brazilian adolescents (12–17 years old) in the southern countryside. Madeira et al., using the homeostasis model assessment for insulin resistance (HOMA-IR), confirmed the presence of MS in Brazilian children aged 2–11 years; this condition is also confirmed by Sentalin et al.^11^ in Brazilian schoolchildren between 6–8 years old in the southern region. In addition, a study focused on overweight and obese schoolchildren (2–11 years old) in Rio de Janeiro city showed a 16.4% prevalence of MS. Furthermore, regardless of definition, metabolic disruption is frequent in Brazilian schoolchildren, especially in overweight and obese ones.^11,25^

The OR variables for cardiometabolic risk were evaluated in schoolchildren with high WC (p>80). Regardless of sex, the OR for cardiometabolic risk was more than sixfold higher in schoolchildren with high value of SBP and triglycerides compared to children with healthy values. Moreover, the schoolchildren with elevated TC presented OR for cardiometabolic risk 30-fold higher, when compared to those with adequate TC values. These data reinforce the importance of evaluating lipids profile in children under ten, mainly in boys who presented higher TC in the present study. Corroborating, Ata et al. demonstrated that the peak of TC in pre-adolescent may take place at about 8–10 years old, with higher rates in boys, followed by a decrease during adolescence, then another peak in late adolescence and young adulthood.^26^ Likewise, a cross-sectional study with schoolchildren aged 8–11 years old, performed in Cuba, demonstrated significant correlation between TC and their subfractions (HDL and LDL) with hypertension.^27,28^

The WC has emerged as an index of pediatric adiposity by predicting fat mass as much as or better than BMI.^27^ Also, increased WC in children and adolescents, regardless of BMI, is a predictor of insulin resistance and is associated with elevated cardiovascular risk factors.^27,29^ The WC p>90 is a good variable to estimate excess of android fat in Brazilian children between 8–9 years old, as demonstrated by Filgueiras et al.^20^ However, the cutoff to WC in children are influenced by sex and ethnicity.^29,30,31^

As recently proposed by Santos et al., the WC p>75 can be considered a cutoff for the risk of obesity in Brazilian children aged 6–8 years^14^. In our study, the Spearman correlation demonstrated that WC p>80 was more strictly related to metabolic and pressure alterations than the Z score of BMI. Confirming Spearman data, in the PCA, we demonstrated that, independently of sex, schoolchildren with WC p>80 presented more elevated values of SBP, DBP, triglycerides, glucose, and TC than the ones with WC p<80. Thus, it’s probable that a new cutoff for WC needs to be elaborated exclusively for Brazilian children.

Taken together, these data reinforce the requirement of more adequate support to characterize cardiometabolic risk or MS in schoolchildren under ten, to avoid non-communicable chronic diseases installation throughout life. In this sense, results from a study among 128 subjects suggested that rapid weight gain during infancy could predict the clustering of metabolic risk factors at 17 years old.^32^ Felisbino-Mendes et al. demonstrated that maternal nutrition state had an impact on the overweight and obesity of Brazilian children.^33^ Moreover, a study recently evaluated metabolic health risk factors in Brazilian schoolchildren between 6–8 years old, showing the contribution of school and family particularities, as well as the health environmental risk factors for obesity in those children. As highlighted by by Magge et al., and Wittcopp and Conroy, more important than establishing the definition of MS in children may be providing guidance to health habits, stimulating weight loss and performing long-term clinical follow-up to lipids and glucose parameters, mainly in overweight and obese children. In this regard, a recent systematic review showed how effective exercise and diet are in order to improve BMI in children under ten years old.^8,34^

Some points could be considered as limitations in our study, particularly the small size of the sample, in addition to the absence of insulin sensitive analysis and demographic profile description, as well as the lack of data on nutritional and physical status from children, variables that would contribute to improve data interpretation.

In conclusion, schoolchildren from the western Parana region showed similar frequency of overweight and obesity if compared to the results found in the southern Brazil, which were above the national median. Besides, among these schoolchildren a narrow relationship was observed between elevated WC and high values of triglycerides, TC, SBP and DBP, all variables that elevated the OR for cardiometabolic risk. It is important to emphasize that the frequency of cardiometabolic risk in schoolchildren aged 5–10 years old was higher than those observed to MS in Brazilian adults, suggesting precocious metabolism disruptions with impacts in cardiovascular functions. Finally, these findings reinforce the importance of early identification of metabolic changes in the pediatric population to avoid non-communicable chronic disease development in adulthood.

## Data Availability

The database that originated the article is available with the corresponding author.
